# AtRD22 and AtUSPL1, Members of the Plant-Specific BURP Domain Family Involved in *Arabidopsis thaliana* Drought Tolerance

**DOI:** 10.1371/journal.pone.0110065

**Published:** 2014-10-15

**Authors:** Vokkaliga Thammegowda Harshavardhan, Le Van Son, Christiane Seiler, Astrid Junker, Kathleen Weigelt-Fischer, Christian Klukas, Thomas Altmann, Nese Sreenivasulu, Helmut Bäumlein, Markus Kuhlmann

**Affiliations:** 1 Research Group Abiotic Stress Genomics, Interdisciplinary Center for Crop Plant Research (IZN), Halle (Saale), Germany, and Leibniz Institute of Plant Genetics and Crop Plant Research (IPK) Gatersleben, Stadt Seeland, OT Gatersleben, Germany; 2 Research Group Gene Regulation, Leibniz Institute of Plant Genetics and Crop Plant Research (IPK) Gatersleben, Stadt Seeland, OT Gatersleben, Germany; 3 National Key Laboratory of Gene Technology, Institute of Biotechnology Vietnam, Academy of Science and Technology, Hanoi, Vietnam; 4 Research Group Heterosis, Leibniz Institute of Plant Genetics and Crop Plant Research (IPK) Gatersleben, Stadt Seeland, OT Gatersleben, Germany; 5 Research Group Image Analysis, Leibniz Institute of Plant Genetics and Crop Plant Research (IPK) Gatersleben, Stadt Seeland, OT Gatersleben, Germany; 6 Grain Quality and Nutrition Center, International Rice Research Institute (IRRI), Metro Manila, Philippines; Institute of Genetics and Developmental Biology, Chinese Academy of Sciences, China

## Abstract

Crop plants are regularly challenged by a range of environmental stresses which typically retard their growth and ultimately compromise economic yield. The stress response involves the reprogramming of approximately 4% of the transcriptome. Here, the behavior of *AtRD22* and *AtUSPL1*, both members of the *Arabidopsis thaliana* BURP (BNM2, USP, RD22 and polygalacturonase isozyme) domain-containing gene family, has been characterized. Both genes are up-regulated as part of the abscisic acid (ABA) mediated moisture stress response. While *AtRD22* transcript was largely restricted to the leaf, that of *AtUSPL1* was more prevalent in the root. As the loss of function of either gene increased the plant's moisture stress tolerance, the implication was that their products act to suppress the drought stress response. In addition to the known involvement of AtUSPL1 in seed development, a further role in stress tolerance was demonstrated. Based on transcriptomic data and phenotype we concluded that the enhanced moisture stress tolerance of the two loss-of-function mutants is a consequence of an enhanced basal defense response.

## Introduction

Abiotic stress factors such as moisture stress, salinity, extreme temperature and variable light intensity can disturb plant metabolism and growth. It has been estimated that crop yield losses due to such stresses lie in the order of 50% [1], so increasing the resilience of crop plants will be an important contributor to yield stability. Abiotic stress affects both photosynthesis and photorespiration, as well as having an impact on the energy and redox status of the plant cell. One of the most damaging products of stress is the group of compounds referred to as reactive oxygen species (ROS). The plant response also includes the induced synthesis of certain enzymes and low molecular weight compounds associated with antioxidant activity, redox regulators, chaperones such as heat shock proteins and late embryogenesis abundant proteins, water and ion transporters, the production of compatible osmolytes to maintain cellular water content and the fine tuning of proteolysis involved in programmed cell death. Numerous attempts, with varying levels of success, have been made to genetically engineer the production of some of these components with a view to enhancing abiotic stress tolerance [2].

The phytohormone abscisic acid (ABA) is involved in the regulation of expression of many stress-responsive genes, although other stress responsive genes are known to be regulated in an ABA-independent manner [3]–[5]. The regulation of several stress-inducible genes is mediated by the interaction of bZIP transcription factors (ABFs) with ABA-response elements (ABRE) in target gene promoters [6].

The *Arabidopsis thaliana* gene *RESPONSIVE TO DEHYDRATION22* (*AtRD22*), originally identified as a gene which responded to dehydration [7], [8], encodes a member of the BURP protein family, members of which share a highly conserved BURP domain at their C terminus [9]), sometimes also referred to as a U domain [10], [11]. BURP proteins appear to be plant-specific; some examples are the *Vicia faba* unknown seed protein (USP) [10], the *Brassica napus* microsporogenesis-specific protein BNM2 [12]–[14], the *Panicum maximum* apomixis-specific protein [15] and the wheat pollen protein RAFFTIN [16]. The soybean genome harbors 23 BURP protein encoding genes, of which 17 are responsive to stress [17]–[20]. The 18 *Populus trichocarpa* BURP family members fall into five recognizable sub-families [21]. There are 15 related genes in maize, one of which is specifically expressed in the root cortex parenchyma [22] while the sorghum genome harbors 11 homologs [23]. The ectopic expression of the *A. thaliana* gene *AtUSPL1* has been shown to distort seed development and to alter the morphology of seed lipid vesicles [24].

*AtRD22* is up-regulated by moisture stress, salinity stress and exogenously supplied ABA [8] and its induction has been used as a marker for abiotic stress [25]–[29]. The heterologous expression in both *A. thaliana* and rice of the soybean gene *GmRD22* enhances salinity stress tolerance [30]. Members of the BURP family are up-regulated by stress in rice [31], soybean [30] and maize [23]. Members of the BURP family have been described in relation to stress conditions. In cotton, an RD22-like protein interacts with an α-expansin and the over expression of both proteins simultaneously promotes growth and fruit weight [32].Remarkably, the expression programmes active during stress response partially overlap with gene expression during early embryogenesis and seed desiccation [24]. Most likely ancient stress response genes have been recruited to protect seed tissue from dehydration stress in drying seeds [33], [34]. Here, a combination of genetic, molecular and physiological approaches has been applied to isolate and characterize T-DNA insertion mutants of *AtRD22* and *AtUSPL1.*

## Materials and Methods

### Plant material and growth conditions

*A. thaliana* seeds (ecotype Col-0 and the three T-DNA insertion lines SALK_146066 *(rd22-1*), WiscDsLox481-484P12 (*rd22-2*) and SALK_022325 (*uspl1*)) obtained from the European Arabidopsis Stock Center (NASC) were stratified before imbibing them for three days at 4°C in the dark. The resulting germinated seedlings were grown in soil for four weeks under 60% relative humidity, a constant temperature of 22°C and under a 16 h photoperiod (light intensity of 120 µmol m^−^^2^ s^−1^). Drought stress was applied by a. active dehydration under low humidity for 1–5 days and b. withholding water for 1–5 days; the relative soil water content was monitored using an HH2 moisture meter (delta-T devices, Cambridge, UK). Moisture stress was also mimicked in two week old seedlings by transferring them for three days on a MS basal medium containing one of 150 mM or 300 mM NaCl, 100 µM ABA, 4% w/v trehalose, 4% w/v sorbitol, 4% w/v glucose, 4% w/v fructose, 4% w/v sucrose, 300 mM mannitol, 15% w/v PEG 6000 or 4% w/v PEG 20000.

**Figure 1:**
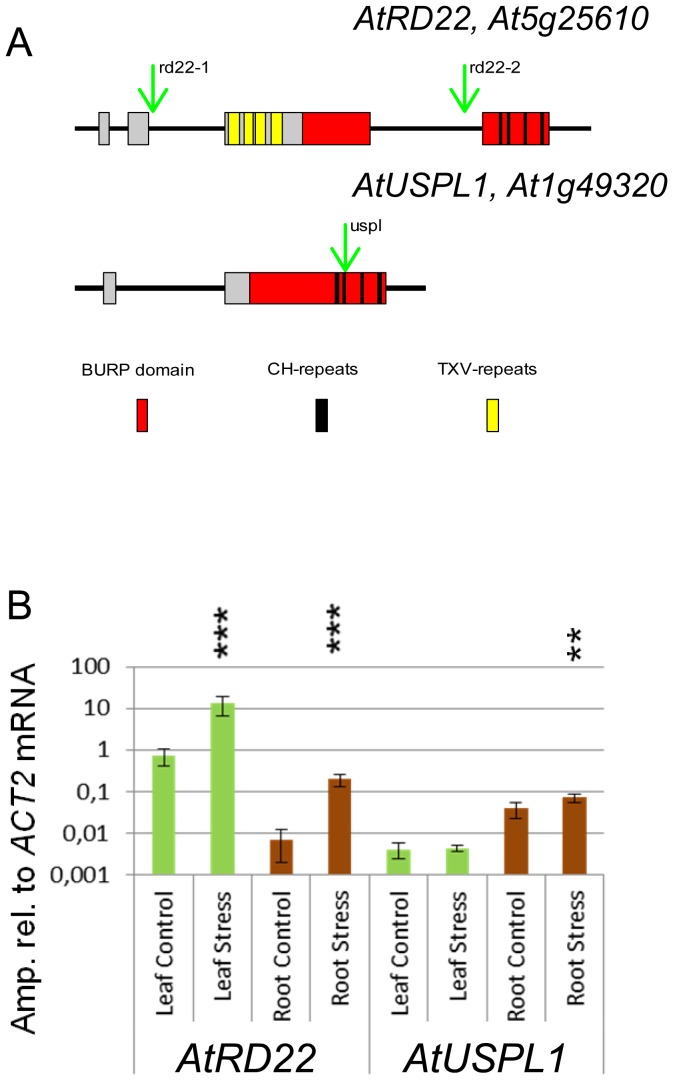
*AtRD22* and *AtUSPL1*, members of the BURP gene family. A. Scheme of BURP-domain containing proteins AtRD22 and AtUSPL1. BURP (named after BNM2, USP, RD22 and Polygalacturonase isozyme) proteins are identified by their C-terminal BURP-domain (red). The BURP domains contain 4 CH-repeats (black). In comparison to AtUSPL1, AtRD22 contains an additional motif, the TXV repeats (yellow), in AtUSPL1 no repetitive domain structure can be found. AGI ID is given in brackets. Position of T-DNA insertions for the used loss-of function mutants is indicated by a green arrow. B. Quantification of *AtRD22* and *AtUSPL1* mRNA in leaves and roots. The bar diagram indicates the amplification of *AtRD22* (left) and *AtUSPL1* mRNA relative to *ACT2* mRNA (amp. rel. to *ACT2* mRNA). Leaf (green) and root (brown) tissue of well watered and drought stressed (2% RWC) plants (N = 5) was analyzed. Asterisks indicate significant differences (T-test: *** p<0.01, ** = p<0.05).

Detailed information is provided in Figure 1/Figure S4. Primers used for genotyping are listed in Table S1. Drought stress was applied by withdrawal of water and relative soil water content [%] was controlled with HH2 moisture meter (delta-T devices Ltd, Cambridge, UK).

### Plasmid construction and plant transformation

Standard protocols [35] were used to prepare plasmids for Gateway Cloning Technology (Invitrogen GmbH, Karlsruhe, Germany). To achieve its ectopic expression, the *AtUSPL1* coding sequence was cloned into the pBENDER GATEWAY vector (a gift from B. Weisshaar, MPI, Cologne).

A 962 bp fragment harboring the *AtUSPL1* promoter was amplified, re-sequenced and inserted into the *Sph*I/*Hin*dIII cloning site of pBIN101 (Clontech) in front of the *uidA GUS* reporter gene. Stable transformation of *A. thaliana* was performed following [36] and subsequent GUS staining to detect transgene expression following [37].

### Automated plant phenotyping and image analysis

After 14 days of pre-cultivation pots (diameter 10 cm) were transferred to an automated phenotyping system composed of a conveyor belt transport system and three imaging chambers for plant imaging in the visible (390–750 nm) and NIR (1400–1510 nm with detection peak at 1452 nm) wavelength ranges as well as capturing fluorescence emission (520–750 nm) as well as a watering and weighing station (LemnaTec 3D Scanalyzer; LemnaTec, GmbH, Aachen, Germany) installed in a climate controlled plant cultivation chamber (environmental conditions: 8 h/16 h night/day, 20/22°C (night/day), relative air humidity of 60% and 240 µmol m^−^^2^ s^−1^ light intensity). Ten blocks (corresponding to ten biological replicates) with each 15 plants (2×5 genotypes under stress conditions, 1×5 under control conditions) were used. Half of the blocks were placed on opposite sides of the cultivation chamber and pots within one block were randomized in order to account for lane or block effects. For reasons of acclimation, plants were grown under control conditions for another week (till day 21 after sowing) before stress application was started. Automated watering was performed every 2^nd^ day to reach defined target values of the pot weight (70% field capacity for control, no watering for soil drought stress). Relative soil water content [%] was controlled in parallel to pot weight with HH2 moisture meter (delta-T devices, Cambridge, UK). The growth rate was calculated as Relative Growth Rate (RGR)  =  (log(LA[tn+1]) - log(LA[tn]))/((tn+1) - tn) following [38]; Leaf area (LA), timepoint n (tn). Statistical significant growth differences were estimated by Welch t-test from values for plant area determined from top view (visual [39]). Detailed information on calculation of NIR intensities and Cameras used are provided in Table S3.

### Estimation of chlorophyll content

Chlorophyll and pheophytin (a chlorophyll degradation product were extracted from the aerial tissue of 6–7 seedlings using a 1∶1 acetone:DMSO mixture, and the absorbance of the extract was recorded at 663, 645 and 553 nm [40] using the UVIKON XS 60/99-90286 spectrophotometer. The experiments comprised three independent replicates. Since the chlorophyll content in the mutant plants grown under non-stressed conditions differed from that present in wild type plants, a relative value was calculated to derive the effect of the stress treatment (Figure S6C). Chlorophyll and pheophytin contents were estimated according to [41], and subjected to a one way ANOVA.

### RNA isolation qRT-PCR and Northern analysis

RNA was isolated using an RNeasy kit (Qiagen, Hilden, Germany). For the purposes of Northern blotting, 10 µg RNA was loaded into each lane of a 1.2% w/v agarose, 15% v/v formaldehyde gel, electrophoresed, then transferred passively onto a Hybond N+ membrane (Amersham, GE Healthcare, Waukesha, USA) using 10× SSC as the transfer buffer. The RNA was cross-linked to the membrane by UV irradiation. An *AtUSPL1* probe was amplified from *A.thaliana* genomic DNA using primer pair USPa/b (sequences given in Table S1) and labeled with α-^32^P dCTP via random priming (Ready Prime Labeling, Pharmacia, GE Healthcare, Waukesha, USA). Membrane/probe hybridizations were carried out in Church hybridization solution [42] at 65°C with pre-hybridization for 6 h and hybridization for 16 h. The membrane was washed for 15 min twice each in 2xSSC, 0.1% SDS, 0.5xSSC, 0.1% SDS and 0.1xSSC, 0.1% SDS at 65°C. Signals were detected and quantified with a Bio-Imaging analyser BAS2000 or X-ray film (Fuji Photo Film Co. Ltd., Tokyo, J). Quantitative real time PCRs (qRT-PCR) were run following [43], in order to assess transcript abundances in leaf and root tissue of six week old plants (five replicate RNA extractions per biological sample, and three technical replications per RNA extract). All primer sequences are given in Table S1.

### RNA Isolation, target synthesis and microarray hybridization

Total RNA was isolated from the aerial tissue of two week old seedlings using the TRIzol reagent (Invitrogen) and RNAeasy columns (Qiagen) (see Figure S7 for detailed information). The integrity of the RNA was monitored by agarose gel electrophoresis, and its concentration estimated using a NanoDrop device (Peqlab, Erlangen, Germany), following the manufacturer's protocol. The integrity of the RNA was further confirmed using an Agilent 2100 Bioanalyzer in conjunction with the RNA 6000 Nano assay (Agilent Technologies, Böblingen, Germany). RNA was processed for use on an Affymetrix Arabidopsis ATH1 Genome Array (ATLAS Niolabs GmbH, Berlin, Germany).

### Microarray data analysis

The ATH1 chip, which assays 22,392 unique genes, was used to contrast the transcriptomes of wild type Col-0, *rd22* and *uspl1* plants grown under both control and moisture stressed conditions. Probe set to target gene mappings were taken from the TAIR Web site: ftp://ftp.arabidopsis.org/home/tair/Microarrays/Affymetrix/affy_ATH1 _array_elements2010-12–20.txt. The microarray experiment and basic data interpretation was performed by ATLAS biolabs GmbH (Berlin, Germany) by GeneChip Operating System (GCOS) 1.4. To ensure reliability of the analyses, each GeneChip experiment was performed with two biological replicates. After logarithmic transformation of the data, the average expression for all experimental samples for this probe set was subtracted from each individual expression value, thus leading to a positive value in case of above-average expression levels and a negative value in case of below-average expression levels. GeneSpring GX software (Agilent) was used to gene-wise normalize the expression data. To identify potentially differentially expressed genes, the fold changes >2 and <2 of expression values were identified. This was done separately for the Col-0 and mutant series at different conditions. For the Col-0 and respective mutant sample, a simple moderated t-test was performed and P values were corrected using the Benjamini and Hochberg [44] false discovery rate control, applying standard limma procedures. Differentially expressed genes, between Col-0 and mutant samples were identified for both conditions using the limma nestedF procedure, applying a significance threshold of 0.5 in combination with Benjamini-Hochberg false-discovery rate control and a minimal log2-fold change value of 2. A functional categorization of the differentially transcribed genes was derived using Mapman software [45].

## Results

### The *BURP* gene family

The *A. thaliana* genome contains five *BURP* family genes; these include both *AtUSPL1 (At1g49320)* and *AtRD22 (At5g25610)* (Figure 1A), but also three genes encoding proteins sharing similarity with the tomato non-catalytic β-subunit of polygalacturonase. The latter have been proposed to be designated as *AtPG1* (*At1g60390*), *AtPG2* (*At1g70370*) and *AtPG3* (*At1g23760*). Based on an alignment of related sequences extracted from various species, the family can be subdivided into eight sub-families [23], according to which *AtRD22* belongs to the ATRD22-like subgroup, *AtUSPL1* to the *BNM2*-like sub-family, and *AtPG1-3* to the PG1β-like subfamily. The *A. thaliana* genome has no representative of either the V-VIII or the VfUSP like sub-groups. The N-terminal regions of *AtPG1-3* each include sequences encoding 21 FXXY–N_9–11_ repeats (of unknown function) [24]. AtRD22 contains four TXV-repeats, while AtUSPL1 has no repetitive features. Based on the domain structure and phenotype this study is restricted to the functional analysis of *AtRD22* and *AtUSPL1*.

### Transcription of *AtRD22* and *AtUSPL1* during development and in response to stress

Some *Arabidopsis BURP* genes were described to be preferentially expressed in early embryogenesis [24], their involvement in stress response was less obvious. An exception of this is *AtRD22*, which was shown previously to be induced under drought treatments [8].

**Figure 2:**
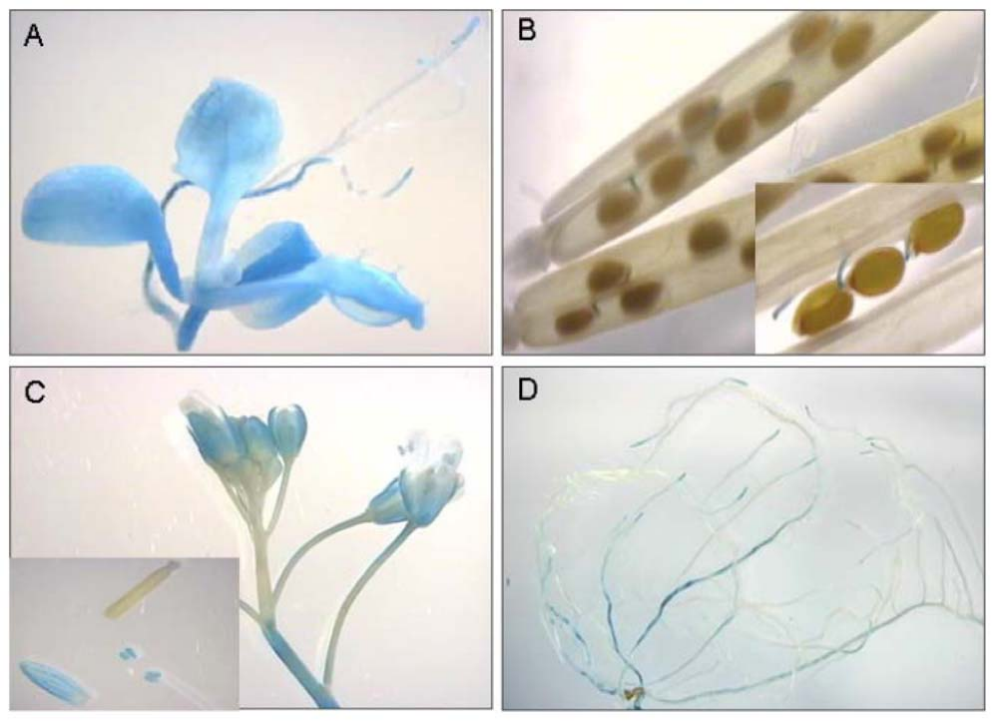
Histochemical *ProAtUSPL1::GUS* activity in transgenic Arabidopsis plants. The *AtUSPL1* promoter activity was determined by histochemical localisation of GUS activity derived from the transgenic *ProATUSPL1::GUS* reporter gene. Activity indicated by blue colour can be seen in A) seedling; B) in funiculus of mature seeds; C) in flowers and stems; and D) in roots.

The transcription profiles of *AtRD22* and *AtUSPL1* were recovered from the Genevestigator database ([46] Figure S1A) and verified using qRT-PCR and Northern blotting. Archival microarray data suggested that while *AtRD22* transcript is abundant throughout plant development in the aerial part of the plant, that of *AtUSPL1* is low and is restricted to the root. In non-stressed plants, *AtRD22* transcription is highest in the leaf, and particularly so in the guard cells [46]. *AtUSPL1* transcript is most abundant in the root (Figure 1B, Figure S1B), but is also detectable in the aerial part of the plant early in development. In the root, *AtUSPL1* transcription is stimulated by exposure to either mannitol or NaCl, as is that of *AtRD22* in the leaf [47]. Here, moisture stress up-regulated *AtRD22* transcription was detected, particularly in the leaf. *AtUSPL1* transcript was detectable in the root, and its level was enhanced by the imposition of moisture stress (Figure 1B). *AtUSPL1* expression was also assayed by tracking GUS expression produced by the *pAtUSPL1::GUS* transgene. GUS activity was detected in the young leaf, in the hypocotyl and in the stem (Figure 2A). In the silique, its expression was only detectable in the mature seed funiculum (Figure 2B), while in the developing flower and stem, a low level of expression was observed (Figure 2C). GUS activity was especially strong in the root tip (Figure 2D). Compared to the strong *AtRD22* promoter activity in aerial parts of the plant [26], *ProAtUSPL1* shows strong transcriptional activity in the root tissue.

According to *in silico* data, treatment with ABA strongly induces *AtRD22* in the leaf, and *AtUSPL1* responds similarly in the root. *AtRD22* is also inducible by exposure to mannitol, glucose, nitrate, high levels of illumination, high temperature and salinity (Figure S2). The gene is down-regulated when the plant is treated with paclobutrazol, an inhibitor of gibberellin synthesis, as well as with cycloheximide, a general inhibitor of protein synthesis, and with syringolin, an inhibitor of cell proliferation.

### The behavior of *AtRD22* and *AtUSPL1* T-DNA insertion mutants

In wild type plants, the expression of the two selected *BURP*-gene family members was confirmed by quantitative RT real-time PCR (Figure 1B) and microarray analysis of the aerial part and among them *AtRD22* transcript is abundant under stress treatments (Figure S3). The loss of function mutants achieved by T-DNA insertions were analysed for characterization of *AtRD22 a*nd *AtUSPL1* genes in the functional context of drought stress tolerance (Figure S4A). For *AtRD22* two independent T-DNA insertion alleles were used: *rd22-1* and *rd22-2*. For *AtUSPL1* the mutant line *uspl1* was used. To analyse the functional loss of both members of the BURP-gene family the *rd22-1/uspl1* double mutant was analysed. In the two analysed T-DNA insertion lines *rd22-1* and *uspl1* no mRNA of the respective gene was detectable by semi-quantitative PCR and Northern analysis (Figure S4B), which is corroborated by microarray analysis (Figure S4C). Therefore we assume that the used T-DNA mutants represent a loss of function mutation of the respective gene.

### Loss of AtRD22 and AtUSPL1 lead to enhanced drought tolerance

**Figure 3:**
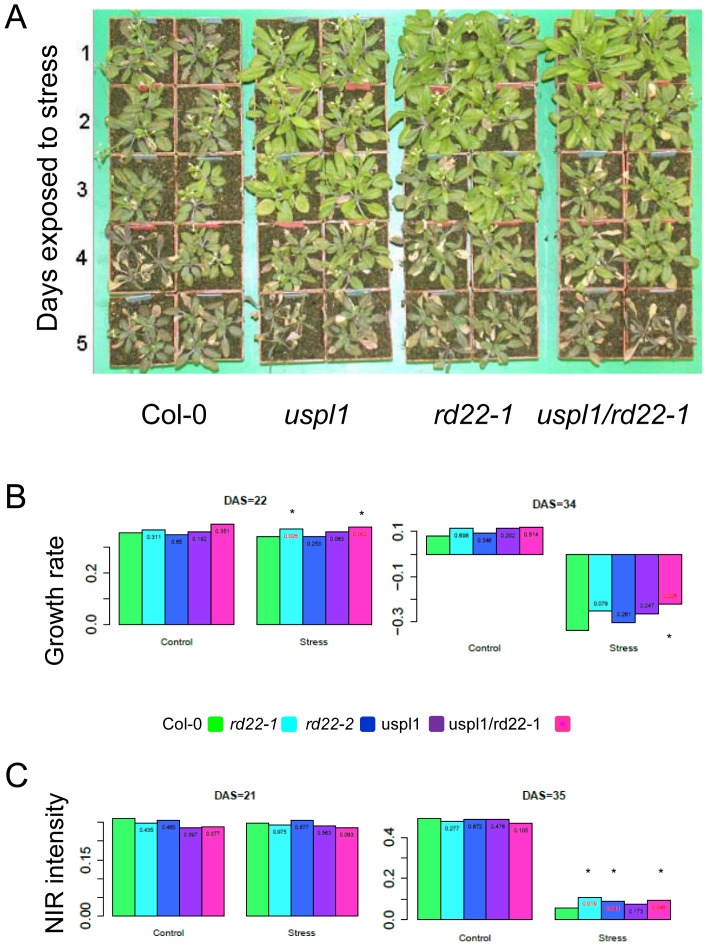
Influence of drought stress treatment on single and double loss of function mutants. A) Four weeks old wild type (Col-0), single and double mutant plants (*rd22-1* and *uspl1, rd22-1/uspl1*) were drought stressed for 1–5 days before they were returned to climate chamber conditions. Time of stress treatment in days is indicated left. B) Growth rates of plants under control and drought stress conditions. Bars indicate the growth rates at 22 days after sowing (DAS) for early phase of drought stress and 34 for the late phase of drought stress. For application of drought stress stop of watering started at 21 DAS. Wild type (Col-0): green bar; *rd22-1*: bright blue bar; *rd22-2*: dark blue bar; *uspl1*: purple bar; *rd22-1/uspl1*pink bar. Original data: Figure S5 B, Table S2, Asterisks indicate significant differences (p<0.01). C) NIR reflection as a water content-related parameter. Bars indicate the NIR intensity at 21 days after sowing (DAS) for start of experiment and at 35 DAS for the end of experiment. Wild type (Col-0): green bar; *rd22-1*: bright blue bar; *rd22-2*: dark blue bar; *uspl1*: purple bar; *rd22-1/uspl1*pink bar. N_control_ = 5, N_stress_ = 10 plants. Original data in Table S3.

The response of the loss-of-function *AtRD22 a*nd *AtUSPL1* T-DNA insertion mutants to moisture stress is summarized in Figure 3 and Figure S4/S5. Two independent *AtRD22* (*rd22-1* and *rd22-2*) and one *AtUSPL1* (*uspl1*) mutants were analysed, along with the *rd22-1/uspl1* double mutant. In both *rd22-1* and *uspl1,* transcript of the mutated gene was not detected based on either semi-quantitative PCR or Northern blotting (Figure S4B), corroborating the prediction of microarray analysis (Figure S4C). When subjected to moisture stress for 2–3 days, wild type plants became discolored as a result of an accumulation of anthocyanin and their growth ceased, whereas both the single and double mutant plants remained green and showed no evidence of any growth retardation (Figure 3A). Exposure to a longer period of moisture stress discriminated between wild type and the mutants in a similar manner (Figure S5A).

### Detection of plant growth via automated phenotyping

To quantify the drought response of the mutant plants an automated phenotyping platform (LemnaTec) was used. The growth of 30 plants of control (Col-0), *rd22-1*, *rd22-2*, *uspl1* and *rd22-1*/*uspl1* each under defined control and drought stress condition was analysed. Drought stress was applied from 21 DAS by complete withdrawal of water. To monitor the growth of the plants, leaf area was estimated from top view images ([39], [48], Figure S5B, Table S2) and used for calculation of relative growth rates [38]. An elevated growth rate of the *rd22-1* single mutant plant as well as for the double mutant plants compared to wild type could be found in the early phase of the experiment (22 DAS, Figure 3B). The first significant drought related difference in growth rate was detectable after 7 days without watering (28 DAS, Figure S5B). A significant difference in the growth rate of the *rd22/uspl1* double mutant plants compared to the wild type plants was detectable almost throughout the entire experiment initially from day 24 till day 32 after sowing under stress conditions (Figure 3B, Figure S5B).

Plant senescence as consequence of the drought stress was monitored by quantifying the ratio of yellow to green pixels. Leaves of the mutant plants *rd22-1*, *uspl1* and the double mutant showed a lower accumulation of yellow stained material, indicative for reduced senescence (33 DAS, Figure S5C). In order to analyse the relative water status of the plants the reflected near-infrared (NIR) radiation (1450 nm) from leaves was detected [49]. NIR intensity is calculated as 1 – NIR reflectance and illustrates the relation to the water content of the leaves. The drought stress related decrease of the NIR intensity was detectable at 33 DAS (Table S3). All genotypes analysed showed a similar level of detectable NIR reflectance in the beginning of the experiment and continuously under control conditions. At the end of the drought stress exposure *rd22* and *rd22/uspl1* double mutant plants showed a higher NIR intensity, indicating higher water content in the leaves (Figure 3C).

### Plant growth response to salinity and osmotic stress and exposure to ABA

To further investigate the role of *AtRD22* and *AtUSPL1* during salinity stress and correlating responses on the transcriptional level, wild type and mutant plants were grown on plates with the respective treatments. Two-weeks-old seedlings were transferred for three days to MS basal medium containing one of the following stress inducing compounds: 150 mM and 300 mM NaCl, 100 µM ABA, 4% trehalose, 4% sorbitol, 4% glucose, 4% fructose, 4% sucrose, 300 mM manitol, 15% PEG 6000 and 4% PEG 20000. Although these conditions are artificially mimicking drought, they were chosen to achieve a uniform plant response to the stimulus. When exposed to either 150 mM NaCl or 100 µM ABA, only minor signs of stress symptoms were apparent, but in the former case, the growth of both the wild type and single mutant plants was retarded (Figure S6A). Wild type and *rd22-1* plants exhibited the least extent of leaf bleaching, while the double mutant and particularly the *uspl1* single mutant, were more visibly affected. Exposure to the various sugars had only a mild effect on plant growth, but a general tendency was for the mutant plants to be more vigorous than the wild type ones. Therefore NaCl 150 mM conditions were chosen for further transcriptome and chlorophyll analysis.

### Chlorophyll and pheophytin content

**Figure 4:**
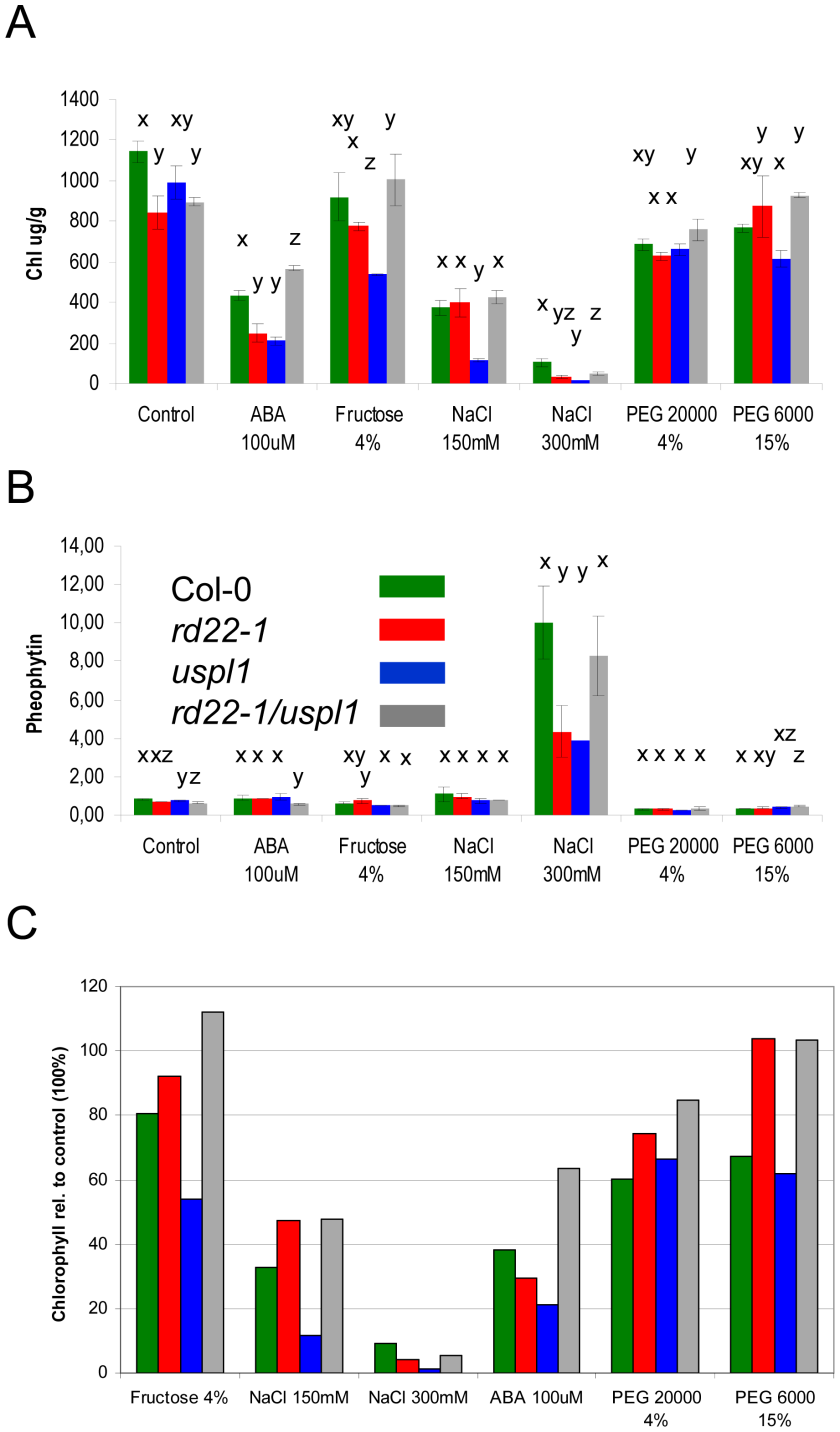
Chlorophyll and pheophytin content in single and double *BURP* mutants. The bars represent the total A) chlorophyll and B) pheophytin content in leaves from wild type (Col-0, green bar), single and double mutant plants (*rd22-1*, red bar, *uspl1,* blue bar and *rd22-1/uspl1*, grey bar). Chlorophyll a and b was determined separately (Figure S6A) and subsumed as total chlorophyll content. Error bar represents standard error. N = 5–6 plants in duplicate. Statistical analysis was performed by oneway ANOVA at alpa  = 0.05 Tukey post hoc test: same letters indicate no difference, different letters indicate significant difference. C) The bars show the total chlorophyll content [%] relative to unstressed control plants.

After a four day exposure to moisture stress, the quantity of chlorophyll a and b in the *rd22-1* mutant and the double mutant leaves was less than that in non-stressed plants (Figure S6C). Since the contents of chlorophyll a and chlorophyll b were strongly correlated with one another, subsequent measurements considered the total chlorophyll content. Pheophytin is a degradation product of chlorophyll, that accumulates during senescence, dark [50] and salt stress [51]. In plants grown in the presence of 300 mM NaCl, the content of chlorophyll (Figure 4A) fell sharply, while that of pheophytin rose (Figure 4B). However when challenged by a lesser level of stress (100 µM ABA or 150 mM NaCl), while the chlorophyll content was reduced, there was no measurable increase in pheophytin content (Figure 4C). Chlorophyll content in the *rd22-1* and *uspl1* mutants was more severely reduced in the presence of either 150 mM NaCl or 100 µM ABA. The *uspl1* mutant was the most compromised genotype with respect to chlorophyll content when the medium was supplemented with fructose, while the *rd22-1* and the double mutant plants out-performed the wild type and *uspl1* mutant plants when the stress was imparted by PEG. To clearly indicate this different chlorophyll degradation in the *rd22-1* single and double mutant plants the amount [%] of total clorophyll is indicated relative to the amount of clorophyll at control conditions (Figure 4C). Compared to the amount of chlorophyll under control conditions the *rd22-1* and *uspl1* mutant plants show the strongest reduction upon 150 mM NaCl and 100 µM ABA. In addition, the reduction of chlorophyll in the *uspl1* mutant with fructose show a major difference compared to the other genotypes. In the *rd22-1* and *rd22-1/uspl1* mutant plants the amount of chlorophyll is higher on fructose, 150 mM NaCl and PEG supplemented media compared to the wild type and *uspl1* mutant plants. Taken into account that the chlorophyll content in the mutant in the unstressed condition varies from the wild type a relative value is indicated to show the change due to the treatment (Figure S6B).

The conclusion was that AtRD22 acts to suppress chlorophyll degradation under moisture stress.

### Transcriptome analysis

The transcriptional responses to exposure to 150 mM NaCl and to 4% w/v trehalose overlapped by about 10% (Figure S6A). The 150 mM NaCl treatment resulted in a changed transcript abundance for 913 of the 22,392 genes represented on the ATH1 chip. Salinity stress and both the trehalose and sorbitol treatments marginally up-regulated *AtRD22*, while the other *BURP* family genes were hardly affected (Figure S6D). The four genes showing the greatest change in transcript abundance between wild type and mutant plants grown under non-stressed conditions (Table 1) were *At4g33720* (encoding CAP, a PR protein under the control of the DREB2A tanscription factor, [52]: up-regulated by 12 fold in both *rd22-1* and *uspl1* compared to wild type); *At5g49700* (encoding a DNA-binding protein associated with moisture stress [53]: up-regulated by 56 fold in *rd22-1* and by 40 fold in *uspl1*); *At2g27550* (encoding ATC, a systemic inhibitor of floral initiation [54]: up-regulated by 23 fold in both *rd22-1* and *uspl1*); and *At2g44380* (encoding a DC1-domain containing protein, involved in the abiotic stress response, [55]: up-regulated by 22 fold in both *rd22-1* and *uspl1*). The full set of differentially transcribed genes detected in response to the various treatments is given in Table S4 (see also Figure S7). A total of 77 genes displayed differential transcription between wild type and *rd22-1* plants grown under non-stressed conditions; 31 out of these are associated with either biotic or abiotic regulatory pathways (Figure S7A) and included eight encoding a peroxidase putatively involved in H_2_O_2_ degradation. The absence of AtUSPL1 only re-programmed 18 genes, of which five (one encoding a peroxidase) are associated with either biotic or abiotic regulatory pathways (Figure S7B). Exposure to 150 mM NaCl resulted in the up-regulation of a number of genes associated with either moisture stress or pathogen defense (Table 2). In *rd22-1*, out of the 764 differentially transcribed genes, 231 fell into one of these two categories (Figure S7C), while in the *uspl1* mutant, only 18 genes were up-regulated, of which five were associated with either the biotic or the abiotic stress response (Figure S7D). In the latter mutant, *AtRD22* was strongly up-regulated in the presence of salinity stress. When the stress was applied by the addition of trehalose to the medium, 55 of the 171 differentially transcribed genes identified in the *rd22-1* mutant were associated with either the abiotic or the biotic stress response (Figure S7E). Over 50% (111 out of 212) of the genes responding to trehalose treatment reacted similarly to salinity stress (Figure S3A, Table S4). The set of common up- regulated genes included the transcription factor genes *AtMYB15* (*At3g23250*) and *WRKY33* (*At2g38470*), *LPT3* and *LPT4* (encoding lipid transporters), *At1g35910* (a putative trehalose-6-phosphate phosphatase), *At1g61120* (terpene synthase 04), *At1g16850* (unknown function) and *At1g78410* (VQ motif-containing protein). Although fewer genes were induced by the trehalose (212) than by the 150 mM NaCl (913) treatment, the conclusion was that the two stress agents must affect a similar class of gene. The response to both stress agents also included the down-regulation of several photosynthesis-related genes.

**Table 1:** Top differential expressed genes in A) *rd22-1* and B) *uspl1* at standard growth conditions in the aerial part of 2 week old seedlings.

A) *rd22*									
Probe Set ID	fold change	Gene	AGI ID	put. Function	Probe Set ID	fold change	Gene	AGI ID	put. Function
257162_s_at	59		At3G24290	amm. transporter	265364_at	−45		At2G13330	Transposon
248564_at	56		At5G49700	DNA binding	246908_at	−40	RD22	At5G25610	Disease Defense
249052_at	47	PDF1.2	At5G44420	Disease Defense	251677_at	−38	ORG3	At3G56980	DNA bind
245276_at	41	ATHB-2	At4G16780		257453_at	−30		At1G65130	hydrolase
265102_at	39		At1G30870	Peroxidase	261684_at	−29		At1G47400	
259276_at	38		At3G01190	PER27 Peroxidase	265665_at	−28		At2G27420	
263096_at	38	AHB1	At2G16060		249645_at	−26	THI2.2	At5G36910	toxin receptor
264567_s_at	37		At1G05250	Peroxidase	266472_at	−26			
254044_at	35	XTR9	At4G25820	hydrolase	254170_at	−22		At4G24430	lyase
254200_at	34		At4G24110		247252_at	−22		At5G64770	

B) *uspl1*									
Probe Set ID	fold change	Gene	AGI ID	put. Function	Probe Set ID	fold change	Gene	AGI ID	put. Function
247718_at	42	LTP4	At5G59310	Lipid transfer	252795_at	−38			
248564_at	40		At5G49700	DNA bind	246948_at	−23	CYP71B12	At5G25130	Cytochr. P450
261226_at	31	ATEXPA11	At1G20190	Expansin	250207_at	−22	CHS	At5G13930	
253667_at	30		At4G30170	peroxidase	256940_at	−19		At3G30720	
252374_at	24	ARR5	At3G48100	transc. Reg.	267168_at	−2		At2G37770	aldo/keto red.
254979_at	23		At4G10550	subtilase					
254832_at	23		At4G12490	protinase inhibitor					
266209_at	23	ATC	At2G27550	A.th centroradialis					
267385_at	22		At2G44380	DC domain					
247717_at	22	LTP3	At5G59320	Lipid transfer					

Complete list of differential expressed genes in Table S4.

**Table 2:** Differential expressed genes in A) *rd22-1* and B) *uspl1* on medium containing 150mM NaCl in the aerial part of 2 week old seedlings.

A) *rd22* on NaCl		
**up regulated genes**		
**Probe Set ID**	**Fold change**	**Gene Symbol**
247215_at	40	**PROPEP3**
266017_at	38	
252984_at	34	**ELI3-2**
252487_at	33	
256627_at	33	
246340_s_at	32	**FAMT**
264005_at	31	AGP2
257206_at	29	
267035_at	26	**AGT3**
259975_at	25	
253872_at	24	RD26
254101_at	24	**AMY1**
266267_at	23	**ATGSTU4**
256245_at	22	**HSP70**
255502_at	21	
247308_at	20	
256436_at	20	
266142_at	11	
256603_at	11	
258791_at	10	**PR4**
264415_at	6	**RAP2.6**
257517_at	4	
258277_at	4	**PAD3**
260919_at	3	
249481_at	2	
**down regulated**		
**Probe Set ID**	**Fold change**	**Gene Symbol**
246366_at	−27	
256772_at	−22	**BGAL1**
250366_at	−21	
261118_at	−20	
261684_at	−20	
267264_at	−17	**SCPL11**
258497_at	−11	**COL2**
261413_at	−11	**PLL5**
246908_at	−7	RD22
247450_at	−2	
261351_at	−2	
266363_at	−1	

B) *uspl1* on NaCl		
up regulated genes		
Probe Set ID	Fold change	Gene Symbol
261684_at	42	
258497_at	21	**COL2**
266363_at	20	
247308_at	18	
247215_at	15	**PROPEP3**
257206_at	14	
249481_at	13	
261118_at	12	
246908_at	11	RD22
256245_at	11	**HSP70**
261351_at	11	
255502_at	11	
250366_at	11	
264415_at	10	**RAP2.6**
266267_at	10	**ATGSTU4**
256225_at	10	
256603_at	4	
258277_at	2	**PAD3**
266017_at	2	
256627_at	1	
267035_at	1	**AGT3**
**down regulated**		
**Probe Set ID**	**Fold change**	**Gene Symbol**
246366_at	−27	
266142_at	−24	
258791_at	−23	**PR4**
267264_at	−20	**SCPL11**
254101_at	−17	**AMY1**
259975_at	−15	
246340_s_at	−15	**FAMT**
256436_at	−15	
256772_at	−14	**BGAL1**
257517_at	−14	
260919_at	−13	
252487_at	−13	
252984_at	−11	**ELI3-2**
264005_at	−11	AGP2
247450_at	−11	
253872_at	−11	RD26
261413_at	−7	**PLL5**

## Discussion

### RD22 and USPL1 have suppressor function during drought stress

*AtRD22* and *AtUSPL1* were transcribed predominantly in, respectively, the leaf and the root. The induction of both genes by moisture stress generated a slight growth reduction. While the induction by moisture stress of *AtRD22* has been noted previously, this was not the case for *AtUSPL1*, most likely due to a concentration on the short-term stress response. Microarray-based transcriptomic analyses have shown that exposure to either NaCl or trehalose induces not only the up-regulation of salinity, trehalose and moisture stress responsive genes, but also the down-regulation of photosynthesis-related genes. The latter is implied by both the induction of leaf chlorosis and senescence and the measured fall in the chlorophyll content of the leaves of stressed plants. The present data confirmed that both salinity and trehalose activate the ABA-mediated moisture stress response. [56]–[58]. The sole *BURP* gene family to be up- regulated in the aerial part of the plant was *AtRD22,* consistent with observations based on the expression of the transgene *pAtRD22::GUS* [26]. The root specificity of *AtUSPL1* expression was confirmed both by the behavior of the *pAtUSPL1::GUS* transgene and by Northern hybridization experiments.

The T-DNA insertion mutants of both *AtRD22* and *AtUSPL1* produced no detectable relevant transcript, so each was taken as a genuine loss-of- function allele. In both cases, a reduced photosynthesis phenotype was exhibited, as reflected by a fall in both chlorophyll content under non-stressed growing conditions and photosynthesis-associated gene transcript abundance in stressed plants. Both under prolonged moisture stress mimicking conditions and actual moisture stress, stress tolerance was enhanced in all of the mutants. The mutants' performance (and particularly that of the double mutant), as measured by either the development of leaf area or by the rate of leaf tissue senescence, showed that they were more tolerant of moisture stress than were wild type plants. NIR intensity appeared to reliably reflect relative water content, which was higher in each of the mutants than in wild type.

Given that photosynthesis is clearly compromised by moisture stress, it was not surprising that the transcription of several photosynthesis-associated genes was altered when the plants were exposed to moisture stress. Although the mutant plants' transcriptomes were indistinguishable from that of the wild type with respect to photosynthesis-related genes, nevertheless their leaves contained less chlorophyll than the leaves of wild type plants raised under non-stressed conditions. The reduction in chlorophyll content in the mutants induced by exposure to either NaCl or PEG was less severe than that in wild type plants. Notably, under moisture stress conditions, the leaves of double mutant plants retained more chlorophyll than those of wild type ones. The retention of chlorophyll can be expected to support a higher rate of photosynthesis, so that less transpiration is required to generate a given quantity of assimilate. Since the plant's capacity to retain its water is improved, its water content under moisture stress conditions was greater (Figure 3C).

The observed up-regulation of peroxidase encoding genes in the mutants suggested a secondary effect of the loss-of-function mutations. The transcriptomic data implied that the oxidative state within the mutant plants differed from that within the wild type, with a knock-on effect on gene expression in both ABA-dependent and ABA-independent pathways [59]. Both the phenotype of the mutants as well as the transcriptional response of both genes suggested that under moisture stress conditions, their products exerted a suppressor function. Similar conclusions have been drawn regarding the effect of mutations of genes encoding SnRK2 [60] the effect of which is an almost complete abolition of the ABA response. In particular, *AtRD22* is not transcribed in these mutants when the growing medium is supplemented with ABA reflecting moisture stress condition. Under normal growing conditions, the *SnRK2* mutants exhibit reduced growth, which (along with plant survival) is improved by the addition of ABA to the growing medium. The inference is that the BURP domain containing proteins AtRD22 and AtUSPL1 act as suppressors of the ABA-mediated moisture stress response.

### RD22 act as suppressor predominantly in the leaf, while USPL1 act in the root

Although both *AtRD22* and *AtUSPL1* exhibited organ-specific transcription, the loss of function of each gene induced a comparable improvement in the plant's tolerance to moisture stress, at least at the level of the vigour of the leaves. The sensitivity of the microarray platform rules out any trans-silencing effects of either T-DNA insertion on other members of the BURP gene family. Since this sort of silencing has been associated with several T-DNA insertion mutations [61], the assumption is that both *AtRD22* and *AtUSP1* are involved in a holistic moisture stress response. The microarray analysis demonstrated that the two loss-of-function mutations resulted in the induction of a partially overlapping set of genes in the aerial part of the plant, even though *AtUSPL1* was transcribed specifically in the root. Not only was the tolerance of moisture stress enhanced in both mutants, but also their chlorophyll content was reduced. As the lack of *AtRD22* and *AtUSP1* led to an enhanced tolerance to moisture stress, the proposed suppressor function of the BURP domain-containing proteins during an episode of moisture stress includes an organ-specific component. Both proteins are part of an ABA mediated moisture stress response pathway, with AtRD22 functioning mainly in the aerial part of the plant and AtUSPL1 in the root.

### Increased drought resistance might be correlated to an increased defence gene response

The transcriptomic analysis of the two mutants revealed an increased transcript abundance compared to the wild type levels with respect to various genes associated with the response to biotic stress (for example, the gene *PDF1.2* was up-regulated by nearly 50 fold). This class of genes was differentially transcribed both under non-stressed and moisture stressed conditions. Several peroxidase encoding genes were also up-regulated. Peroxidases are known to represent an integral component of the plant's hypersensitive response [62]. H_2_O_2_ is also known as signal molecule during the stress response [63], [64]. A loss of control over the production of H_2_O_2_ might result in the described reduction of chlorophyll content [65], [66] and increased water content under drought stress conditions. Such regulation would subsequently lead to better performance of the mutant plants under drought stress.

The up-regulation of peroxidase encoding genes implies a level of linkage between the ABA-mediated moisture stress response and defense against pathogen invasion. Such a connection has been proposed in a suggested model for the function of OCP3, a homeodomain transcription factor. The loss of *OCP3* function results in an enhanced tolerance to moisture stress and at the same time an increased sensitivity to ABA. The abundance of *AtRD22* transcript in the *ocp3* mutant is not different to that in the wild type, and the plant's susceptibility to *Botrytis cinerea* infection is reduced [67]. The *ocp3* mutant was initially identified and named after the phenotype of constitutive over expression of a cationic peroxidase [68]. Here, *AtOCP3* was marginally down regulated when the plants were challenged by NaCl, but there was no transcriptional difference between wild type and either of the two mutants. The implication is that the BURP-containing proteins act to enhance the plant's moisture stress response via the up-regulation of peroxidase encoding genes. In soybean, a direct interaction between RD22 and cell wall-localized peroxidases has been described [69]. The up- regulation of H_2_O_2_ detoxifying enzymes enhances moisture stress tolerance [70] as well as explaining the link with the pathogen defense response.

A reduced chlorophyll content, in conjunction with an elevated level of transcription of defense response genes under non-stressed growing conditions, suggests that the mutant plants are primed to mount a stronger and/or more rapid set of measures to prevent the accumulation of ROS. A change in the accumulation of H_2_O_2_ would not only have an impact on the defense response [71], but also on the response to moisture stress [63], [72]. Due to the overall elevated expression of H_2_O_2_-detoxifying enzymes such responses could be limited, leading to an enhanced drought resistance. Our study provides the first functional approach to investigate *BURP-*domain encoding gene function in addition to the previously published structural comparisons.

## Supporting Information

Figure S1 **Expression profile of the** ***Arabidopsis thaliana BURP*** **gene family.** A) Expression profile of the *Arabidopsis thaliana BURP* gene family. Data obtained from Genevestigator database (Zimmerman *et al.*, 2004). Relative expression of *AtRD22* (red) and *AtUSPL1* (blue) is given for the different developmental stages of Arabidopsis life cycle (left to right: germinating seed, seedling, Young rosette, developed rosette, bolting, young flower, developed flower, flowers and siliques, mature siliques, senescence). B) Expression of *AtUSPL1* confirmed by Northern Blot analysis. Expression of *AtUSPL1* was determined from root, leaf, shoot, young silique and total flower tissue of *Arabidopsis thaliana* (*Col-0*) plants. For equal loading of the RNA samples probing of the membrane with specific probe against housekeeping mRNA of *AtROC1* (rotamase cyclophilin, renamed in *AtCYP1*) was performed. C) Expression of *AtRD22* obtained from Arabidopsis eFP Browser (Winter *et al.*, 2007). The expression of selected stimuli (Cold: 4°C, Osmotic: 300 mM Mannitol, Salt: 150 mM NaCl and Drought: air steam 15 min) is displayed for the aerial as well as the hypogeic part of the plant. *AtRD22* expression is induced in the aerial part of the plant after applying osmotic, salt stress and slightly increased after loss of water due to airstream treatment. D) Expression of *AtUSPL1* obtained from Arabidopsis eFP Browser (Winter *et al.*, 2007). The expression of selected stimuli (Cold: 4°C, Osmotic: 300 mM mannitol, Salt: 150 mM NaCl and Drought: air steam 15 min) is displayed for the aerial as well as the hypogeic part of the plant. *AtUSPL1* expression is induced in the hypogeic part of the plant after applying osmotic, salt stress and slightly increased after loss of water due to airstream treatment.
(TIF)

Figure S2 **Expression of the BURP domain containing gene family in** ***Arabidopsis thaliana*** **.** Expression analysis of *AtRD22* (*At5G25610*) and *AtUSPL1* (*At1G49320*) obtained from Genevestigator database (Zimmerman et al., 2004) displaying induced and reduced expression after different conditions and stresses. Displayed are only changes in expression upon stress/treatment above threefold with a statistic significance p<0.001. Red indicates up-regulation; Green indicates down-regulation.
(TIF)

Figure S3 **Comparison of gene expression in Arabidopsis wild type plants grown on 150 mM NaCl and 4% trehalose supplemented MS medium.** BURP gene family mRNA in *Col-0* under selected stress conditions. Bars indicate the expression pattern obtained by microarray analysis using ATH1 chip: *AtRD22* (red): 246908_at; *AtUSPL1* (blue): 262388_at; *AtPG1* (dark green): 265131_at; *AtPG2* (green): 264277_at; *AtPG3* (bright green): 264315_at. Displayed is the rel. Abundance of mRNA.
(TIF)

Figure S4 **Characterization of BURP mutant plants.** A) Scheme of the *AtRD22* and *AtUSPL1* gene model. In blue the exon-intron structure within the coding region of the respective gene is given. The protein structure refers to Figure 1. In the encoded protein parts are given in aminoacids [aa]. And the size of the fulllenght protein is given below the gene description. Position of T-DNA insertions of used mutant plants are indicated by black lines. Mutant alleles for *rd22*: *rd22-1* (SALK_146066) and *rd22-2* (WiscDsLox481-484P12). Mutant alleles for *uspl1*: *uspl1*: (SALK_022325). The T-DNA insertion line SALK_146066 (*rd22-1*) is based on pROK2 conferring kanamycin resistance and the WiscDsLox481-484P12 (*rd22-2*) is based on pWiscDs-Lox conferring phosphinotricin (BASTA) resistance. *uspl1* T-DNA insertion lines SALK_022325 (referred to as *uspl1*, based on pROK2 conferring kanamycin resistance from Nottingham Arabidopsis Stock Centre) was analyzed. The position of the T-DNA insertion in *At1G49320 (AtUSPL1)* was determined by PCR and subsequent sequencing. The position of the T-DNA insertion are depicted and confirmed by PCR. Double mutant *rd22-1*/*uspl1* line was generated by crossing SALK_146066 and SALK_022325 and identified in the F_3_ generation by PCR. Kanamycin and phosphinotricin resistance of the plants was tested on germination medium (1 MS salts; 10 g/l sucrose) plates with 40 mg/l kanamycin or 20 mg/l glufosinate-ammonium under long day conditions. B) Analysis of used T-DNA insertion mutants. The absence of *AtRD22* and *AtUSPL1* mRNA in homozygous *rd22* and *uspl1* mutant plants was determined by Northern Blot analysis (left) and semi quantitative RT-PCR. C) Analysis of BURP-gene family mRNA in *rd22-1* and *uspl1* mutants by microarray analysis on MS medium. Bars indicate the rel. expression signal obtained by microarray analysis using ATH1 chip from each single experiment: *AtRD22* (red): 246908_at; *AtUSPL1* (blue): 262388_at; *AtPG1* (dark green): 265131_at; *AtPG2* (green): 264277_at; *AtPG3* (bright green): 264315_at.
(TIF)

Figure S5 **Increased drought stress resistance of the** ***rd22*** **and** ***uspl1*** **mutant plants.** A) The plants were drought stressed by withdrawal of water. Top: day 0 (80% RWC in the soil); Bottom: appearance of plants after 8 days without watering. B) Top: Projected area of wild type and mutant plants under control conditions and drought stress (dotted line) obtained by lemnatec phenotyping; Drought stress was started 21 days after sawing (DAS). Middle: Growth rates calculated based on Poorter and Lewis 1986 for individual days. Wild type (Col-0): green line; *rd22-1*: bright blue line; *rd22-2*: dark blue line; *uspl1*: purple line; *rd22-1/uspl1*pink line (+/- s.e.m.). Bottom: Statistical analysis or growth rates at 28 DAS. Asterisks indicate significant differences (p<0.05) between control and stress. C) Estimation of senescence after drought stress. The graph indicates the ration of yellow to green pixels in the plant area of the analysed top view images from day 33. Wild type (Col-0): green bar; *rd22-1*: bright blue bar; *rd22-2*: dark blue bar; *uspl1*: purple bar; *rd22-1/uspl1*: pink bar. N_control_ = 5, N_stress_ =  10 plants. Asterisks indicate significant differences (p<0.05).
(TIF)

Figure S6 **A) Differentially expressed genes (Col-0) between 150 mM NaCl containing medium and standard growth conditions sorted by relation to pathway (Mapman).** Top: Differentially expressed genes (Col-0) between 150 mM NaCl containing medium and standard growth conditions sorted by relation to pathway (Mapman). The different numbers indicate different categories/pathways and are described in the table. Bottom: Differentially expressed genes (Col-0) between standard growth conditions and 4% trehalose containing medium. The bar diagram indicates the percentage of common (red) and inverse (yellow) regulated genes upon salt (grey) and sugar (blue) treatment. Approximately half of the genes induced by 4% trehalose treatment are also reacting on 150 mM NaCl treatment. B) Influence of ABA and NaCl on single and double loss of function mutants. Growth phenotypes of wild type (Col-0), single and double mutant plants (*rd22-1* and *uspl1, rd22-1/uspl1*) on standard MS-medium and 150 mM NaCl, 300 mM NaCl and 100 µM ABA supplemented MS-Medium. Two week old seedlings were transferred for 3 days to the MS basal and supplemented medium. C) Chlorophyll and pheophytin content of wild type and *rd22-1*, *uspl1* and *rd22-1/uspl1* mutant plants on different supplemented media. Two week old seedlings were transferred to the MS basal + one of the following stress treatments for 4 days: 100 µM ABA, 4% fructose, 150 mM NaCl, 300 mM NaCl, 4% PEG 20000 and 15% PEG 6000. From Top to bottom: Chlorophyll a and b content. The error bar represents standard error. Content was estimated from 5-6 plants in duplicate. Statistical analysis was performed by oneway ANOVA at alpa  = 0.05 Tukey post hoc test: same letters indicate no difference, different letters indicate significant difference. Chlorophyll a and b and pheophytin content [%] relative to unstressed control plants.
(TIF)

Figure S7 **Differentially expressed genes in the** ***rd22-1*** **and** ***uspl1*** **plants grown on control, 150 mM NaCl and 4% trehalose containing medium.** List of used categories is given in Figure 3 A. Top: Display of top regulated category (Bin 20, biotic and abiotic stress pathways) of differential regulated genes. Bottom: Overview of all differentially regulated genes mapped to categories (Bins) by MAPMAN. For RNA extraction tissue was grinded in liquid nitrogen and the homogenized powder was added to 1 ml TRIZOL and incubated at RT for 5 min. Samples were centrifuged at 10.000 rpm for 10 min and the supernatant was transferred to a new tube. 200 µl of chloroform were added and incubated at room temperature for 2–3 min. Samples were again centrifuged as described above and the aqueous supernatant was transferred to the QIAshredder column and centrifuged for 30 s at 10000 rpm. 350 µl of RLT buffer (plus β-mercaptoethanol) and 250 µl of absolute ethanol were added to the flow-through and passed through an RNAeasy spin column. All the following steps were performed as described in the manufacturer's protocol followed by in-column DNAse digestion. A) Schematic display of differentially expressed genes in *rd22-1* by MAPMAN. 31 out of 77 differential regulated genes are mapping to biotic and abiotic stress pathways. B) Schematic display of differentially expressed genes in *uspl1* by MAPMAN. 5 out of 18 differential regulated genes are mapping to biotic and abiotic stress pathways. C) Schematic display of differentially expressed genes in *rd22-1* on 150 mM NaCl by MAPMAN. 231 out of 764 differentially regulated genes are mapping to biotic and abiotic stress pathways. D) Schematic display of differentially expressed genes in *uspl1* on 150 mM NaCl by MAPMAN. 7 out of 12 differentially regulated genes are mapping to biotic and abiotic stress pathways. E) Schematic display of differentially expressed genes in *rd22* on 150 mM NaCl by MAPMAN. 55 out of 171 differentially regulated genes are mapping to biotic and abiotic stress pathways.
(TIF)

Table S1 **Primer information.**
(XLS)

Table S2 **Average projected plant area (mm2) from top images.**
(XLSX)

Table S3 **Average near-infrared intensity as observed from top images, obtained using a Nir 300 camera from VDS Vosskühler (now Allied Vision Technologies).** High values indicate relative high water content.
(XLSX)

Table S4 **List of differentially expressed genes.**
(XLSX)
