# Supplementary figures and images for: AtRD22 and AtUSPL1, Members of the Plant-Specific BURP Domain Family Involved in Arabidopsis thaliana Drought Tolerance
Source: PLoS One. 2014 Oct 15;9(10):e110065. doi: 10.1371/journal.pone.0110065 (PMC4198191; doi:10.1371/journal.pone.0110065)

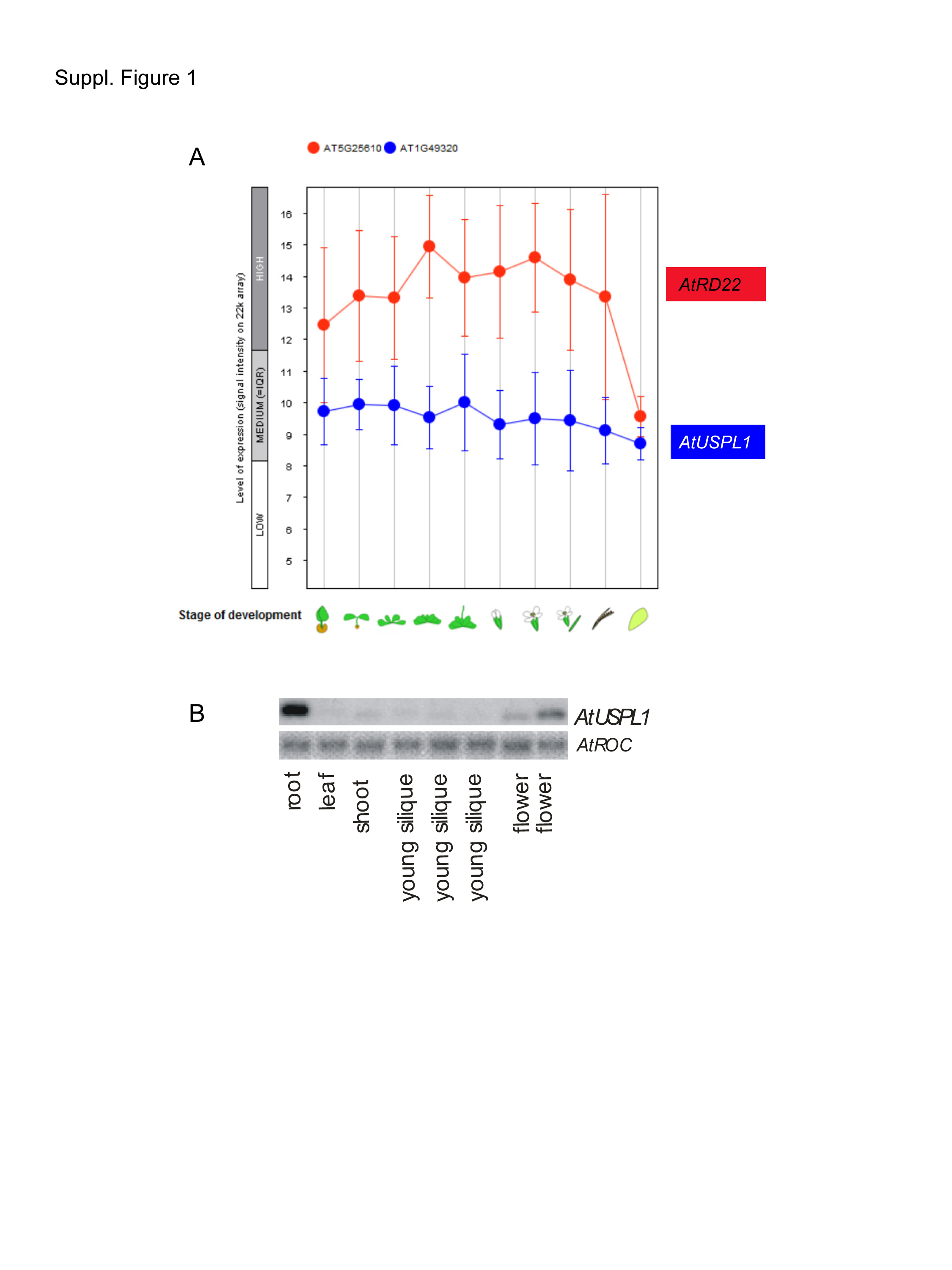

Supplement: Figure S1 — Expression profile of the Arabidopsis thaliana BURP gene family. A) Expression profile of the Arabidopsis thaliana BURP gene family. Data obtained from Genevestigator database (Zimmerman et al., 2004). Relative expression of AtRD22 (red) and AtUSPL1 (blue) is given for the different developmental stages of Arabidopsis life cycle (left to right: germinating seed, seedling, Young rosette, developed rosette, bolting, young flower, developed flower, flowers and siliques, mature siliques, senescence). B) Expression of AtUSPL1 confirmed by Northern Blot analysis. Expression of AtUSPL1 was determined from root, leaf, shoot, young silique and total flower tissue of Arabidopsis thaliana (Col-0) plants. For equal loading of the RNA samples probing of the membrane with specific probe against housekeeping mRNA of AtROC1 (rotamase cyclophilin, renamed in AtCYP1) was performed. C) Expression of AtRD22 obtained from Arabidopsis eFP Browser (Winter et al., 2007). The expression of selected stimuli (Cold: 4°C, Osmotic: 300 mM Mannitol, Salt: 150 mM NaCl and Drought: air steam 15 min) is displayed for the aerial as well as the hypogeic part of the plant. AtRD22 expression is induced in the aerial part of the plant after applying osmotic, salt stress and slightly increased after loss of water due to airstream treatment. D) Expression of AtUSPL1 obtained from Arabidopsis eFP Browser (Winter et al., 2007). The expression of selected stimuli (Cold: 4°C, Osmotic: 300 mM mannitol, Salt: 150 mM NaCl and Drought: air steam 15 min) is displayed for the aerial as well as the hypogeic part of the plant. AtUSPL1 expression is induced in the hypogeic part of the plant after applying osmotic, salt stress and slightly increased after loss of water due to airstream treatment. (TIF) [file pone.0110065.s001.tif]

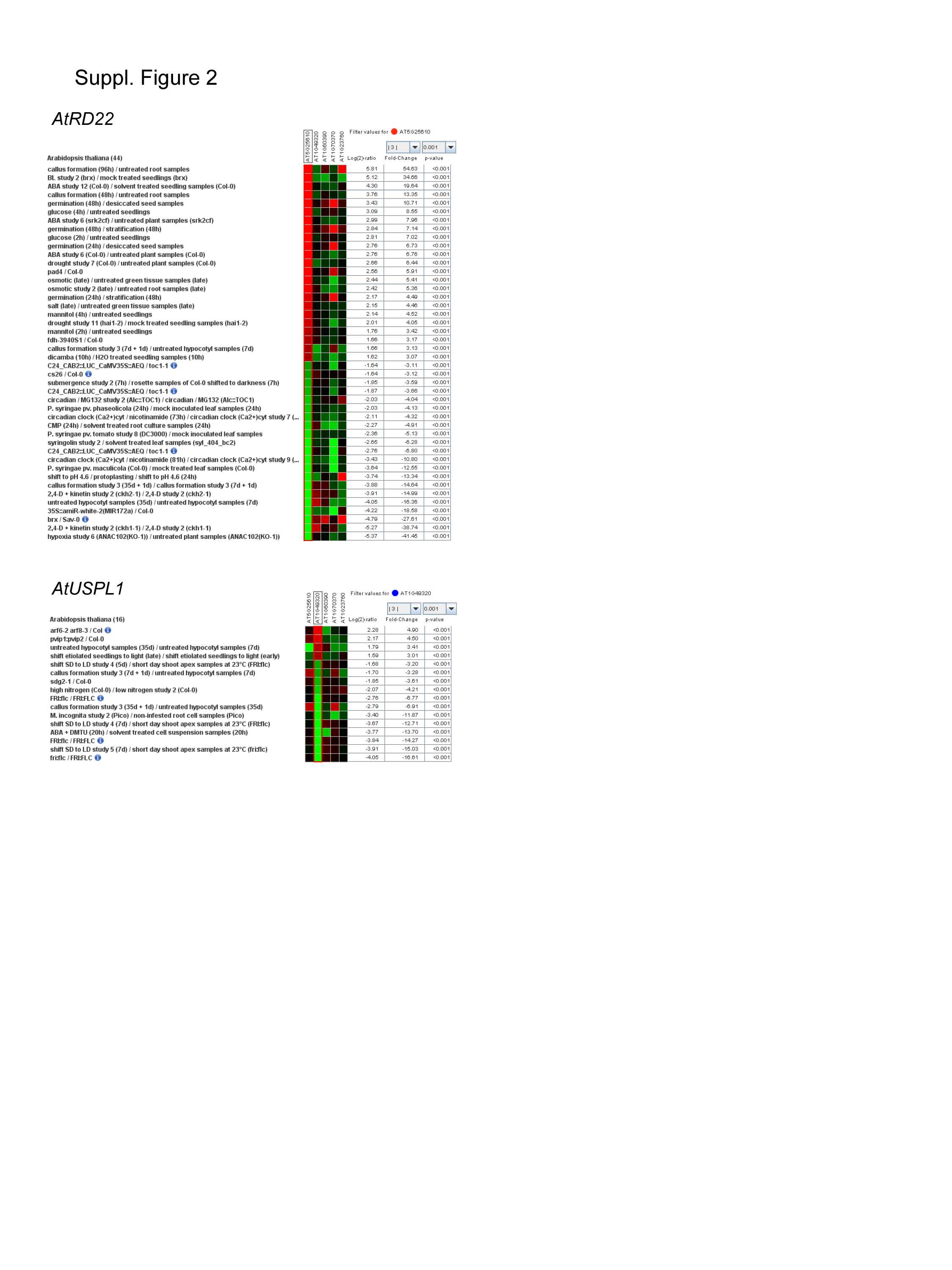

Supplement: Figure S2 — Expression of the BURP domain containing gene family in Arabidopsis thaliana . Expression analysis of AtRD22 (At5G25610) and AtUSPL1 (At1G49320) obtained from Genevestigator database (Zimmerman et al., 2004) displaying induced and reduced expression after different conditions and stresses. Displayed are only changes in expression upon stress/treatment above threefold with a statistic significance p<0.001. Red indicates up-regulation; Green indicates down-regulation. (TIF) [file pone.0110065.s002.tif]

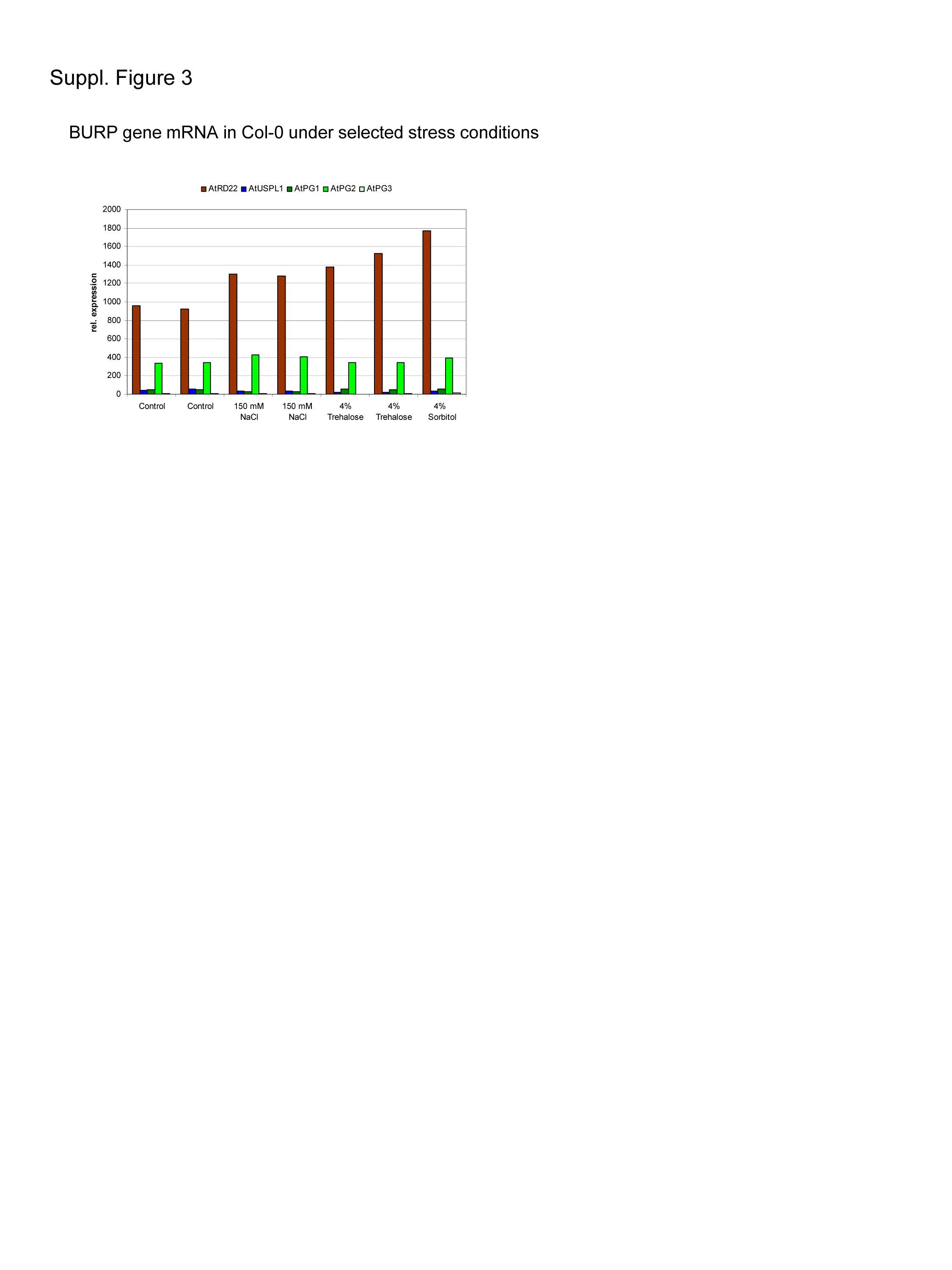

Supplement: Figure S3 — Comparison of gene expression in Arabidopsis wild type plants grown on 150 mM NaCl and 4% trehalose supplemented MS medium. BURP gene family mRNA in Col-0 under selected stress conditions. Bars indicate the expression pattern obtained by microarray analysis using ATH1 chip: AtRD22 (red): 246908_at; AtUSPL1 (blue): 262388_at; AtPG1 (dark green): 265131_at; AtPG2 (green): 264277_at; AtPG3 (bright green): 264315_at. Displayed is the rel. Abundance of mRNA. (TIF) [file pone.0110065.s003.tif]

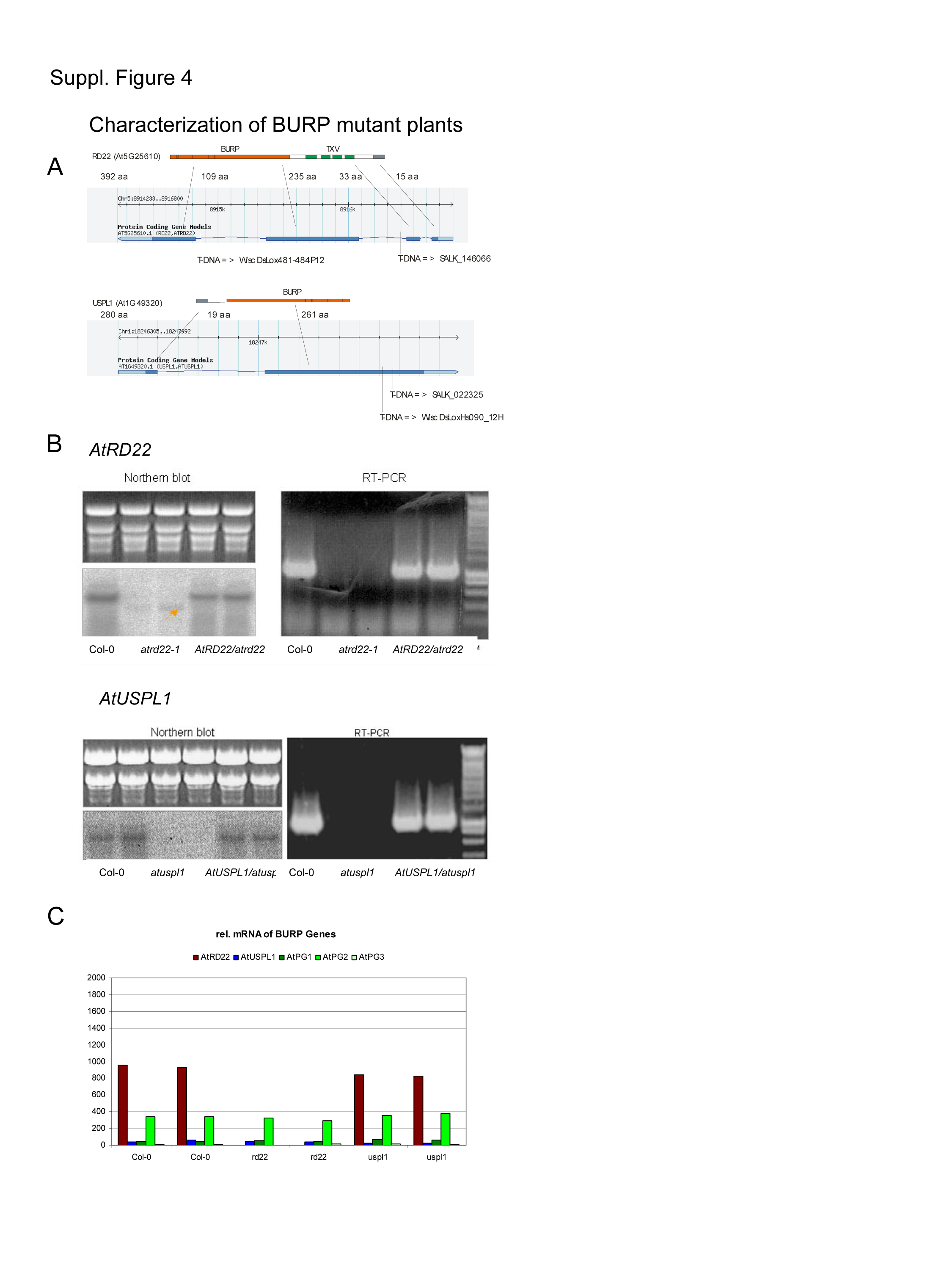

Supplement: Figure S4 — Characterization of BURP mutant plants. A) Scheme of the AtRD22 and AtUSPL1 gene model. In blue the exon-intron structure within the coding region of the respective gene is given. The protein structure refers to Figure 1. In the encoded protein parts are given in aminoacids [aa]. And the size of the fulllenght protein is given below the gene description. Position of T-DNA insertions of used mutant plants are indicated by black lines. Mutant alleles for rd22: rd22-1 (SALK_146066) and rd22-2 (WiscDsLox481-484P12). Mutant alleles for uspl1: uspl1: (SALK_022325). The T-DNA insertion line SALK_146066 (rd22-1) is based on pROK2 conferring kanamycin resistance and the WiscDsLox481-484P12 (rd22-2) is based on pWiscDs-Lox conferring phosphinotricin (BASTA) resistance. uspl1 T-DNA insertion lines SALK_022325 (referred to as uspl1, based on pROK2 conferring kanamycin resistance from Nottingham Arabidopsis Stock Centre) was analyzed. The position of the T-DNA insertion in At1G49320 (AtUSPL1) was determined by PCR and subsequent sequencing. The position of the T-DNA insertion are depicted and confirmed by PCR. Double mutant rd22-1/uspl1 line was generated by crossing SALK_146066 and SALK_022325 and identified in the F3 generation by PCR. Kanamycin and phosphinotricin resistance of the plants was tested on germination medium (1 MS salts; 10 g/l sucrose) plates with 40 mg/l kanamycin or 20 mg/l glufosinate-ammonium under long day conditions. B) Analysis of used T-DNA insertion mutants. The absence of AtRD22 and AtUSPL1 mRNA in homozygous rd22 and uspl1 mutant plants was determined by Northern Blot analysis (left) and semi quantitative RT-PCR. C) Analysis of BURP-gene family mRNA in rd22-1 and uspl1 mutants by microarray analysis on MS medium. Bars indicate the rel. expression signal obtained by microarray analysis using ATH1 chip from each single experiment: AtRD22 (red): 246908_at; AtUSPL1 (blue): 262388_at; AtPG1 (dark green): 265131_at; AtPG2 (green): 264277_at; AtPG3 (bright g [file pone.0110065.s004.tif]

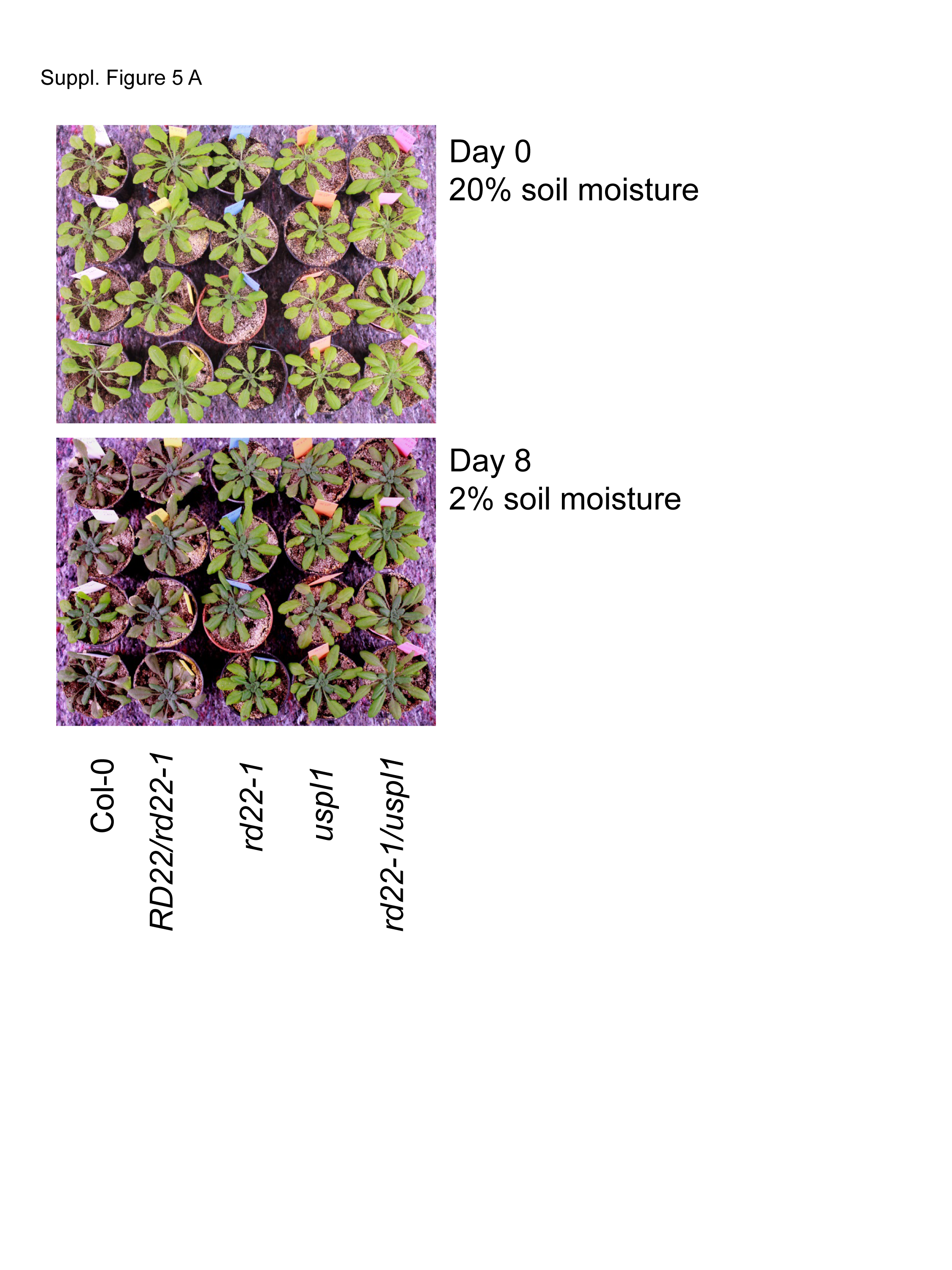

Supplement: Figure S5 — Increased drought stress resistance of the rd22 and uspl1 mutant plants. A) The plants were drought stressed by withdrawal of water. Top: day 0 (80% RWC in the soil); Bottom: appearance of plants after 8 days without watering. B) Top: Projected area of wild type and mutant plants under control conditions and drought stress (dotted line) obtained by lemnatec phenotyping; Drought stress was started 21 days after sawing (DAS). Middle: Growth rates calculated based on Poorter and Lewis 1986 for individual days. Wild type (Col-0): green line; rd22-1: bright blue line; rd22-2: dark blue line; uspl1: purple line; rd22-1/uspl1pink line (+/- s.e.m.). Bottom: Statistical analysis or growth rates at 28 DAS. Asterisks indicate significant differences (p<0.05) between control and stress. C) Estimation of senescence after drought stress. The graph indicates the ration of yellow to green pixels in the plant area of the analysed top view images from day 33. Wild type (Col-0): green bar; rd22-1: bright blue bar; rd22-2: dark blue bar; uspl1: purple bar; rd22-1/uspl1: pink bar. Ncontrol = 5, Nstress = 10 plants. Asterisks indicate significant differences (p<0.05). (TIF) [file pone.0110065.s005.tif]
